# Genome-wide *Escherichia coli* stress response and improved tolerance towards industrially relevant chemicals

**DOI:** 10.1186/s12934-016-0577-5

**Published:** 2016-10-13

**Authors:** Martin Holm Rau, Patricia Calero, Rebecca M. Lennen, Katherine S. Long, Alex T. Nielsen

**Affiliations:** Novo Nordisk Foundation Center for Biosustainability, Technical University of Denmark, Hørsholm, Denmark

**Keywords:** Biochemicals, Chemical stress, Tolerance, Systems biology, Transcription analysis, Tn-seq, *E. coli*

## Abstract

**Background:**

Economically viable biobased production of bulk chemicals and biofuels typically requires high product titers. During microbial bioconversion this often leads to product toxicity, and tolerance is therefore a critical element in the engineering of production strains.

**Results:**

Here, a systems biology approach was employed to understand the chemical stress response of *Escherichia coli*, including a genome-wide screen for mutants with increased fitness during chemical stress. Twelve chemicals with significant production potential were selected, consisting of organic solvent-like chemicals (butanol, hydroxy-γ-butyrolactone, 1,4-butanediol, furfural), organic acids (acetate, itaconic acid, levulinic acid, succinic acid), amino acids (serine, threonine) and membrane-intercalating chemicals (decanoic acid, geraniol). The transcriptional response towards these chemicals revealed large overlaps of transcription changes within and between chemical groups, with functions such as energy metabolism, stress response, membrane modification, transporters and iron metabolism being affected. Regulon enrichment analysis identified key regulators likely mediating the transcriptional response, including CRP, RpoS, OmpR, ArcA, Fur and GadX. These regulators, the genes within their regulons and the above mentioned cellular functions therefore constitute potential targets for increasing *E. coli* chemical tolerance. Fitness determination of genome-wide transposon mutants (Tn-seq) subjected to the same chemical stress identified 294 enriched and 336 depleted mutants and experimental validation revealed up to 60 % increase in mutant growth rates. Mutants enriched in several conditions contained, among others, insertions in genes of the Mar-Sox-Rob regulon as well as transcription and translation related gene functions.

**Conclusions:**

The combination of the transcriptional response and mutant screening provides general targets that can increase tolerance towards not only single, but multiple chemicals.

**Electronic supplementary material:**

The online version of this article (doi:10.1186/s12934-016-0577-5) contains supplementary material, which is available to authorized users.

## Background

The finite supply of fossil fuels and their environmental impact has stimulated the development of alternative biobased solutions for production of chemicals derived from the petrochemical industry [1–3]. This industry relies on the conversion of fossil fuels into a limited number of chemicals that serve as building blocks for the production of a wealth of other chemicals. Likewise, a shift to a widespread biobased chemical production will result in the generation of building blocks employed as precursors for the production of other chemicals [4]. Several potential building blocks have been proposed and include organic acids, amino acids, sugar alcohols, aldehydes and organic solvents, while commodity chemicals of interest include, among others, biofuels and polymer precursors [4–6]. The microbial production of such building blocks and commodity chemicals necessitates high titers to be economically sustainable. However, high titer poses a challenge for the microorganisms, as product toxicity is a likely consequence. Accordingly the development of efficient production strains has to be accompanied by engineering strains with high product tolerance [7, 8]. In addition to product toxicity, inhibitors present in biomass feedstock constitute another hindrance for microbial biomass conversion [9, 10].

Several strategies for engineering improved chemical tolerance exist that address the problem from different angles. One is improved chemical tolerance by rational engineering employing e.g. systems biology to understand the stress condition. Other strategies utilize mutant library screening, e.g. transposon or plasmid based, or natural selection by evolution to identify targets that improve tolerance [7, 11]. In *E. coli* these strategies have been employed towards a variety of chemicals, including but not limited to the investigation of the transcriptional response towards butanol [12], isobutanol [13], acetate [14], octanoic acid [15], produced free fatty acids (FFAs) [16] and furfural [17]. Rational engineering attempts include improving tolerance towards butanol [18, 19], produced FFAs [20], and furfural [21, 22], while screening strategies have been employed towards butanol [23], acetate [24], and furfural stresses [25]. Evolution based selection strategies have been implemented towards butanol [26–30], isobutanol [31–33], acetate [34, 35], octanoic acid [36], serine [37] and succinic acid stresses [38]. Commonly, tolerance identification strategies are performed with exogenous addition of chemical with the rationale that most chemicals will have a cellular impact by diffusion, transport or membrane interaction. Moreover high extracellular titers are needed for an economically viable production.

In this work a combination of systems biology and screening approaches were employed to identify potential targets for improving chemical tolerance in *E. coli.* A thorough investigation of the chemical stress responses to 12 chemicals, including future building blocks and commodity chemicals, was undertaken to find shared features of the different transcriptional responses these chemicals impose on *E. coli*. To this end shared enrichments of functional and regulatory gene sets were identified. These genes are part of the chemical stress response and therefore candidates for tolerance engineering. In addition, a transposon mutant library sequencing analysis (Tn-seq) was carried out with the aim of identifying target genes conferring a tolerance improvement towards the selected chemicals. The inclusion of 12 chemicals in this study enabled the identification of targets that improve tolerance towards not just a single chemical but towards groups of chemicals with shared properties.

## Results

### Selection of chemicals and growth conditions

**Fig. 1:**
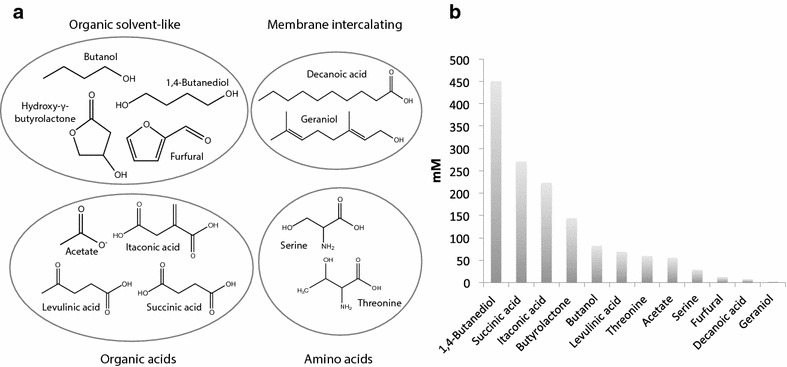
**a** Structures of the chemicals employed in this study. Twelve chemicals were included that based on properties can be divided into four groups. **b** Concentrations (mM) causing a 33 % growth rate reduction

The *E. coli* transcriptome has been investigated under chemical stress conditions with 12 different compounds (Fig. 1a) in order to understand the cellular response and potentially infer the cellular effects of the imposed stress. Commonalities between transcriptional responses could provide understanding of general chemical stress as well as targets for tolerance engineering towards not just single compounds but groups of compounds with similar properties. A main criterion for the selection of chemicals was their potential for biobased production. Hence, most of the chosen chemicals were selected from the predicted top 30 chemical building blocks for future biobased production defined in a report by the US Department of Energy [4]. The chemicals on this list were evaluated based on the criteria of having a sufficient level of inhibition and solubility, while avoiding more than two compounds with similar structures. Ultimately seven chemicals from this list were selected including hydroxy-γ-butyrolactone (Byr), furfural (Furf), itaconic acid (Ita), levulinic acid (Lev), serine (Ser), succinic acid (Suc), and threonine (Thre). Five other chemicals were selected based on other criteria including acetate (Ace), a common inhibitor during fermentation; butanol (But), a high-potential biofuel; 1,4-butanediol (Diol), a polyester precursor; geraniol (Ger), a biofuel and fragrance ingredient and decanoic acid (Deca). The latter belongs to the group of medium-chain fatty acids that can act as biofuel precursors [39, 40]. The selected chemicals have been grouped into four categories to reflect similarities in their chemical properties and expected cellular effects (Fig. 1a). Four chemicals (butanol, hydroxy-γ-butyrolactone, 1,4-butanediol, furfural) are to varying extents utilized as solvents and can therefore be described as organic solvent-like chemicals. Another four chemicals (acetate, itaconic acid, levulinic acid, succinic acid) are referred to as acids, two (l-serine, l-threonine) are amino acids, and two (decanoic acid, geraniol) are labeled membrane-intercalating based on their expected interaction with the bacterial membrane.

The study was designed to elucidate the inhibitory effects of the chemicals and investigate the chemical-specific transcriptional response without triggering a general severe stress response. To this end, concentrations were chosen that causes a 33 % reduction of the exponential growth rate, corresponding to a doubling time increase from 60 min to around 90 min. The resultant inhibitory concentrations of the chemicals span a wide range from just above 1 mM for geraniol to around 450 mM for 1,4-butanediol (Fig. 1b). The two membrane-intercalating chemicals have the lowest inhibitory concentrations. For the chemicals with higher inhibitory concentrations an osmotic stress is expected in addition to chemical stress. The cells were grown in minimal medium in triplicates for each condition and chemicals added during the exponential growth phase. Following 1 h of chemical stress, the cells were harvested for transcriptome profiling by RNA-seq.

### Global transcriptional response towards the selected chemicals

**Fig. 2:**
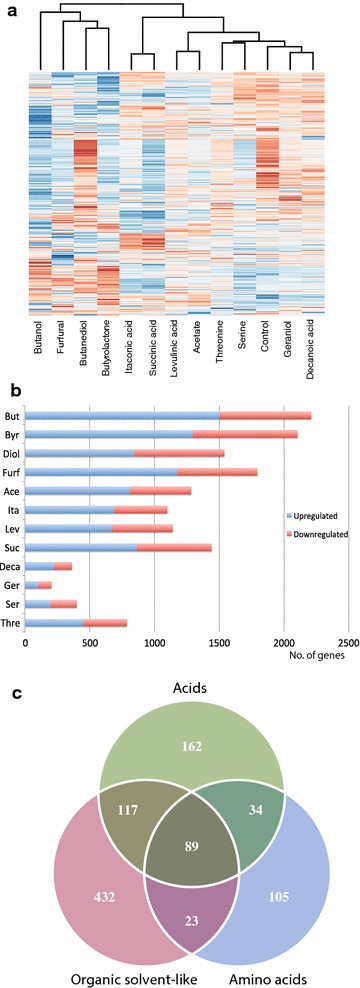
**a** Heatmap and hierarchical clustering of transcriptomic data for all conditions. Only significant genes are included. *Red* and *blue* signifies high and low relative expression, respectively. **b** Quantity of significantly upregulated (*blue*) and downregulated (*red*) genes for each condition. **c** Venn diagram displaying the overlap of significant genes within and between three of the chemical groups. Only significant genes changed in the same direction within or between groups are included

The chemical stress conditions elicit varied and extensive transcriptional responses. An overview of the global transcriptional profile for each chemical (Fig. 2a) reveals that clustering of the conditions correlates well with the chemical properties of the compounds. The organic solvents constitute a subcluster as do the amino acids and membrane-intercalating chemicals, while the acids are clustered into two adjacent groups. Although the clustering suggests similarities within the chemical groups, examination of branch lengths indicates that differences also exist. For example, relatively long branch distances exist among the organic solvents. The acids cluster as dicarboxylic acids (itaconic acid and succinic acid) and monocarboxylic acids (levulinic acid and acetate), but this could also reflect the relatively high sodium ion concentration required for neutralization of the dicarboxylic acids.

The transcriptional response towards many of the chemicals is extensive (Fig. 2b) (Additional file 1). It is clear that both organic solvents and acids have an immense impact on the cell and the transcriptome even at a relatively low 33 % growth rate reduction and consequently have a broad effect on many cellular processes. In contrast, the amino acid and membrane-intercalating groups appear to have a more specific effect on the cell. For the latter group the cellular effects of the chemicals could be more immediate upon addition, possibly via rapid membrane interaction and destabilization, and therefore less pronounced after 1 h leading to a lower transcriptional response. For geraniol, evaporation and adherence of the chemical to the culture flask could also be issues.

On the basis of the clustering there is good agreement between the grouping of chemicals based on their chemical properties and their actual transcriptional profiles. Further inspection also reveals a large overlap of genes displaying differential expression in the same direction within the defined groups (Fig. 2c). There are 661 common differentially expressed genes between all the organic solvent conditions (either upregulated or downregulated in all), 402 between the acid conditions, 251 between the amino acid conditions and 121 for the membrane-intercalating conditions. Based on the average number of significant genes for each organic solvent condition one would expect 148 genes to be differentially expressed in all four conditions by chance (without taking expression direction into account) instead of 661, thus demonstrating a clear overrepresentation of overlapping genes. A substantial overlap is not only present within chemical groups but also between them. There are 206 common differentially expressed genes between the acids and organic solvents and 89 between organic solvents, acids and amino acids. This indicates that similar responses exist not only between chemicals with similar properties but also to some degree between chemicals with different properties.

### Functional expression overlap in multiple chemical stress conditions

Numerous gene expression changes are shared between multiple chemical stress conditions. Functional categories of many of the shared differentially expressed genes are presented in Tables 1 and 2. Many upregulated genes relate to energy metabolism, including genes involved in anaerobic respiration, fermentative pathways, glycolysis, the pentose phosphate pathway and the electron transport chain. The downregulation of motility and chemotaxis genes is a shared feature for all conditions and constitutes the majority of the 89 gene expression changes that are shared between the three chemical groups included in Fig. 2c.

**Table 1:** Upregulated gene functions in multiple conditions

	Gene, protein or function	No. of genes	Chemicals^a^											
			But	Byr	Diol	Furf	Ace	Ita	Lev	Suc	Deca	Ger	Ser	Thre
Anaerobic respiration	Hydrogenase 1	6	x	x		x	x	x	x	x				
	Hydrogenase 3	9	x	x		x								
	Hydrogenase 4	11	x	x		x	x	x		x				
	Nitric oxide reductase	3	x	x		x		x	x	x				
	Nitrate reductase 2	5	x	x	x	x	x		x	x				x
	Glycerol dehydrogenase	3	x	x		x	x		x	x				x
	Lactate dehydrogenase	2	x	x	x	x	x		x		x			
	Fumarate dehydrogenase	4	x	x	x	x	x	x	x	x				x
Fermentation	Pyruvate formate lyase	3	x	x	x	x								
	Acetate (*pta, ackA*)	2	x		x		x	x		x			x	x
	Alcohol dehydrogenase	2	x	x	x	x	x	x	x	x				x
	Lactate dehydrogenase	1	x	x	x	x	x							x
Electron transport chain	NADH dehydrogenase I	13	x	x		x								
	NADH dehydrogenase II	1	x	x		x								x
	Cytochrome d	2	x	x	x	x	x						x	x
	Menaquinol biosynthesis	6	x	x			x	x	x	x				
	Ubiquinol biosynthesis	8	x	x		x								
Acid resistance	Acid fitness island	12		x			x		x		x			x
	Glutamate decarb. (*gadAB*)	2		x	x		x	x	x		x	x		x
	Antiporters (*gadC, adiC, cadA*)	3	x	x	x	x	x	x	x					
	Cyclopropane fatty acid (*cfa*)	1	x	x	x	x	x	x	x	x	x	x		x
Oxidative stress	*sodC, katE*	2	x	x	x	x	x	x	x	x				
Carbon metabolism	Pentose phosphate pathway	8	x	x	x	x	x			x				
	Glycolysis	11	x	x	x	x	x							
Ethanolamine	Metabolism (*eut*)	12	x	x	x	x								
Thiamine	Biosynthesis	6	x	x	x	x		x	x	x				x
Membrane	Colanic acid synthesis	20	x	x^b^		x^b^	x	x		x				
Iron sulphur cluster	Isc	4	x	x	x	x	x		x			x	x	x
	Suf	6		x	x		x	x	x	x	x			
Potassium	Transporter (*kdp*)	5	x	x		x	x	x		x	x	x		
Quorum sensing autoinducer	Synthesis	2	x	x	x	x	x	x	x	x		x		x
	Transport	4	x	x	x	x	x		x					
Sigma factors	*rpoS*	1	x	x	x	x	x	x	x	x	x	x		x
	*rpoE*	1	x	x	x	x	x	x		x				x
	*rpoH*	1	x	x	x	x				x	x	x		x
	*rpoD*	1	x	x	x	x	x							x
	*rpoN*	1	x	x	x	x								

^a^An x denotes that half or more of the genes within a functional categories are differentially expressed

^b^For butyrolactone and furfural 7 and 9, respectively, out of 20 genes are significant

**Table 2:** Downregulated gene functions in multiple conditions

Function	Gene, protein or function	No. of genes	Chemicals^a^											
			But	Byr	Diol	Furf	Ace	Ita	Lev	Suc	Deca	Ger	Ser	Thre
Iron uptake	Ferrichrome transport	4	x	x	x	x								
	Enterobactin transport	6	x	x		x								x
	Enterobactin synthesis	7	x	x	x	x								x
	Ferrous iron transport	3	x	x	x	x		x		x				x
	Ferric citrate transport	4	x	x	x	x								x
Sugar transport	Glucose transport	1		x	x	x			x	x			x	x
	Mannose transport	3		x			x	x	x	x			x	x
	Galactose transport	3	x	x	x		x	x	x	x	x	x	x	x
	Galactitol transport	3		x	x	x	x	x	x	x	x		x	x
	Maltose transport	6		x		x	x	x	x	x	x	x	x	x
ATP	Synthase	9		x	x	x	x	x	x	x				x
Membrane	ECA antigen synthesis	10	x	x	x									
	O antigen synthesis	10	x	x	x									
Copper	Efflux (*cus*)	4				x		x	x	x		x	x	x
Motility	Flagella	35	x	x	x	x	x	x	x	x		x	x	x
	Chemotaxis	6	x	x	x	x	x	x	x	x	x	x	x	x
Biosynthesis	Amino acid, nucleotide, polyamine		x	x	x	x								

^a^An x denotes that half or more of the genes within a functional category are differentially expressed

The organic solvent and acid conditions display the most widespread changes in the functional categories presented in Tables 1 and 2. The genes associated with anaerobic energy generation are upregulated for organic solvents and acids, whereas the genes associated with general energy generation are only upregulated in the presence of organic solvents. The concomitant increases in glucose metabolism, electron transport chain and biosynthesis genes for the ubiquinol electron carrier are consistent. However, the increase in anaerobic energy metabolism is extensive and more surprising. Most anaerobic electron donor and acceptor genes are changed as are genes of fermentation enzymes and the electron carrier menaquinol. Taken together the data indicate that challenge with the organic solvent and acid chemicals, and especially the former, leads to a stress that generates a need for increased energy production. Another shared feature for organic solvents and acid conditions is the upregulation of genes involved in acid resistance including the acid fitness island, glutamate decarboxylase, amino acid antiporters and membrane modification genes. For the acid conditions this could be expected, but less so for the organic solvent conditions.

Genes of iron-sulfur cluster assembly systems (isc and suf) are upregulated for both acids and organic solvent conditions, indicating either oxidative stress or iron limitation. The upregulation of iron transport genes for organic solvent conditions however indicates the opposite; an excess of iron. Genes encoding other transporters such as potassium, copper and sugar transporters are also affected. Upregulation of genes involved in potassium uptake is present in both organic solvent and acid conditions and normally indicates an increase in osmotic stress. Sugar transporter genes are downregulated in both acid and organic solvent conditions including the primary glucose transporter gene (*ptsG*), a downregulation which opposes the upregulation of glycolytic genes. Other membrane modifications include genes involved in the synthesis of M antigen which is upregulated for most organic solvents and acid conditions, whereas genes involved in enterobacterial common antigen (ECA) and O antigen synthesis are downregulated for organic solvents. The M antigen, or colanic acid, is associated with biofilm formation and can be induced by osmotic and detergent stress, possibly explaining why it is upregulated here. The ECA is a glycolipid and has been related to resistance towards bile acids and short-chain fatty acids [41].

Many sigma factors have increased expression in multiple conditions. This is especially the case for the organic solvent conditions, where five of the main sigma factors (*rpoS, rpoE, rpoH, rpoD, rpoN*) exhibit significant increases in expression, providing an explanation for the vast transcriptional changes observed in these conditions. Some of the above mentioned expression changes during organic solvent stress are likely the effect of an increase in *rpoS* and *rpoE* expression (Table 1). However, upon examination of the transcriptional changes, footprints of *rpoH* and *rpoN* upregulation are less obvious. For instance, upregulation of some of the well-known chaperone genes regulated by RpoH (*dnaJK*, *groEL*) is not observed. For the acid conditions it is mainly *rpoS* and *rpoE* that are increased, matching well with the displayed expression changes. The observed expression changes for quorum sensing autoinducer related genes in the acid and organic solvent conditions could be a consequence of increased *rpoS* expression, although other explanations cannot be ruled out.

Metabolism and biosynthesis genes in particular are also affected in many conditions. Apart from those already mentioned, many biosynthesis genes are downregulated for organic solvents, including many encoding amino acid synthesis enzymes (glutamate, glutamine, tryptophan, methionine, arginine), nucleotide biosynthesis enzymes and polyamine biosynthesis and transport proteins. Accordingly, it seems that glucose is shunted towards energy generation rather than anabolism. However some biosynthesis genes are also upregulated, including ethanolamine for organic solvents, thiamine for most conditions and branched chain amino acid synthesis genes for various conditions. Phosphatidylethanolamine is one of the most abundant phospholipids in the membrane and the upregulation of ethanolamine metabolism genes could be a consequence of recycling of the phospholipid from within the cell or from other cells.

### Differential regulator activity inferred from regulon enrichment

The response to a stimulus is mediated by the cellular regulatory network. In an attempt to elucidate the origins of the widespread transcriptional changes observed in response to chemical stress, the potential changes in regulator activity have been examined. These changes in activity may also provide clues to the direct cellular effects imposed by a chemical. Changes in regulator activities were determined by statistical analysis of gene enrichment of the regulon of each regulator using the R package piano [42]. This approach is similar to standard gene enrichment analyses where functional categories are employed instead.

**Fig. 3:**
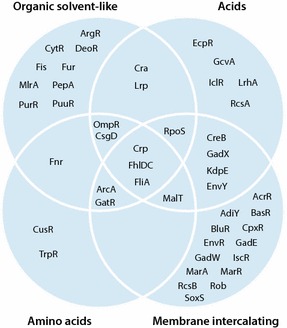
Venn diagram displaying regulators with significantly enriched regulons within and between chemical groups. Regulons enriched in 3 out of 4 conditions for acids and organic solvents and 2 out of 2 conditions for amino acids and membrane-intercalating chemical groups are included as enriched in the respective chemical group

Many regulons are enriched for each condition as would be expected based on the vast transcriptional changes, and considerable overlaps within and between chemical groups are also present (Fig. 3). The regulons of flagellum transcription factors FlhDC and FliA are enriched for all chemicals and the regulon expression changes demonstrate a reduced activity of these two regulators leading to the downregulation of flagella and chemotaxis genes. The other universally enriched regulon for all chemical groups is that of catabolite repression protein (CRP), a major regulator of metabolism and many other processes in the cell. Out of the 509 genes in the regulon between 200 and 300 of these are differentially expressed in e.g. the organic solvent conditions. In Tables 1 and 2 the likely genes regulated by CRP are the sugar transport genes. The enrichment of the GatR and MalT regulons (Fig. 3) and therefore putative change in activity of these regulators are likely an effect of the changes in CRP activity.

The OmpR regulon is altered in all chemical groups except the membrane-intercalating group. OmpR activity is dependent on changes in osmolarity and it is likely that either the osmotic or the membrane interacting effect of the chemicals could elicit increased OmpR activation. OmpR positively regulates the *flhC* and *flhD* genes and it is therefore possible that the downregulation of flagellar genes are caused by OmpR activation. The fact that flagellar genes are downregulated in all conditions and the OmpR regulon is not enriched in all, does not exclude this, as *ompR* is upregulated transcriptionally in the two conditions (membrane-intercalating) where the regulon is not enriched. The absence of OmpR regulon enrichment in this group is due to genes in the *csgDEFG* operon being part of the regulon and not exhibiting differential expression here. CsgD activation is in turn a probable effect of OmpR activation.

ArcA and FNR, whose regulons are enriched for three groups (organic solvents, membrane intercalating and amino acids) and two groups (organic solvents and amino acids) of chemicals respectively, respond to anaerobic conditions and regulate genes involved in respiration and fermentation. Here, the majority of anaerobic respiration and fermentation genes presented in Tables 1 and 2 seem to be upregulated by these two regulators. Yet, the footprint of ArcA and FNR regulation is not pronounced for all genes within the regulons as quite a few display no differential expression or even expression in the opposite direction, possibly due to the effects of other regulators. One of the most enriched conditions is butanol while other conditions have a less pronounced enrichment in the correct directions.

This characteristic is also present in the regulons of Cra and Lrp as these genes are enriched in acid and organic solvent conditions. However for many of the genes, the direction of expression changes are not consistent with the effect of the regulator. Hence, these two regulators may not have changed activity. The differential expression of many of the genes in the regulons could rather be an effect of other regulators. The enrichment of the Fur regulon in the organic solvent conditions however corresponds well with the observed downregulation of iron transporters.

### Genome-wide screening for genes involved in chemical stress tolerance

Understanding the cellular chemical stress response can indirectly provide information and clues towards improving tolerance. However, experimental approaches such as Tn-seq provide a more direct generation of targets for tolerance improvement. Tn-seq is a selection-based approach in which the distribution of a population of genome-wide transposon mutants is measured by sequencing and counting insertions in each gene before and after selective experimental conditions. It thereby provides a fitness estimate of each mutant in the experimental condition based on its pre- and post-selection abundance within the population. Here, a population of approximately 60,000 mutants were subjected to chemical stress at higher concentrations leading to doubling times of approximately 3 h, compared to a control with a doubling time of 1 h.

**Fig. 4:**
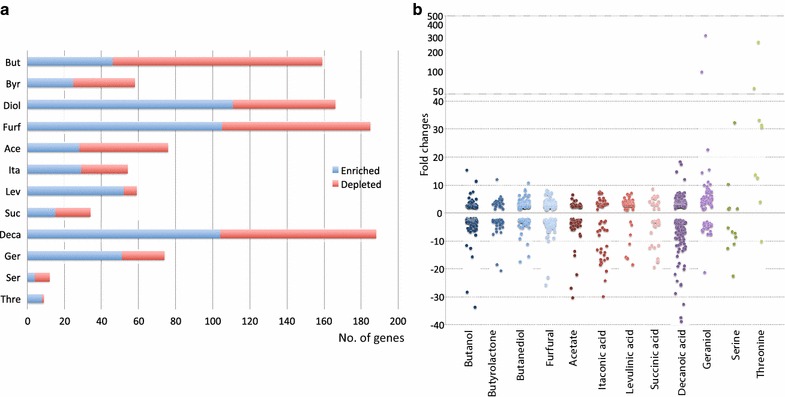
Significantly enriched or depleted Tn-seq transposon insertion mutants. **a** Quantity of significantly enriched (*blue*) or depleted (*red*) gene transposon insertion mutants for each stress condition. **b** Fold changes of significant genes for which transposon mutants are either enriched (positive fold changes) or depleted (negative fold changes)

In all conditions there were mutants with increased and decreased fitness (Fig. 4a) and there is a slight trend towards more insertions conferring a fitness advantage rather than a fitness impairment. The organic solvents, apart from butyrolactone, again had a large number of genes differentially enriched or depleted in transposon insertions and the amino acid conditions likewise had a low number. By contrast, the membrane-intercalating conditions, especially decanoic acid, had relatively high numbers of genes with significant abundance changes in transposon insertions. The quantity of significant genes could be an indicator of the number of potential targets for tolerance improvement. Hence many targets could exist towards organic solvent and decanoic acid stresses while the potential low number of enriched genes for amino acids could reflect the specificity of this stress. Additional file 2 contains a list of all genes enriched and depleted in transposon insertions in all conditions.

The actual fold changes of transposon mutants are generally uniformly distributed within the range of ±tenfold (Fig. 4b). A fold change of ten would here on average correspond to a growth rate increase of 39 %. Specific enriched insertion mutants are presented in Table 3. These consist of the top-two genes for each condition as well as other significant genes from Tn-seq that were tested experimentally for improved tolerance towards the relevant condition. In order to investigate the validity of Tn-seq predictions, a total of 35 gene insertion mutants from the Keio collection of single gene knockout mutants [43] were tested. Eight insertion mutants (80 %) of the tested top-two genes with highest fold changes displayed improved tolerance while in total nineteen insertion mutants (54 %) displayed improved tolerance. Although there is not a direct correlation between the quantitative values of fold change and growth rate change, most beneficial insertions predicted by Tn-seq are true positives. Consequently the majority of Tn-seq predictions are expected to be correct and therefore provide a valuable list of targets for tolerance improvement.

**Table 3:** Fold changes of enriched transposon mutants in Tn-seq and growth rate increases of corresponding gene deletion mutants

	Gene^a^	Fold change^b^	Growth rate % (P value)	Function
Butanol	*rob* ^c^	15.3	32.8 (2E−3)	Right oriC-binding transcriptional activator
	*rraA* ^c^	11.4	8.2 (4E−2)	Ribonuclease E (RNase E) inhibitor protein
	*srmB*	7.0	16.3 (9E−3)	ATP-dependent RNA helicase
	*yecR*	4.9	NS	Lipoprotein, function unknown
	*ydaE*	4.1	NS	Rac prophage; conserved protein
	*aroL*	2.5	18.0 (1E−2)	Shikimate kinase II
	*iscR*	2.4	20.4 (2E−2)	Isc operon transcriptional repressor
Butyro-lactone	*marB* ^c^	12.0	30.3 (2E−2)	Mar operon regulator
	*flxA* ^c^	5.9	ND	Qin prophage; uncharacterized protein
	*cspC*	5.1	30.9 (1E−2)	Stress protein, member of the CspA-family
	*sdhD*	4.2	−40.4 (3E−3)	Succinate dehydrogenase
	*brnQ*	2.8	NS	Branched-chain amino acid transport system 2 carrier protein
	*alx*	1.9	32.9 (8E−3)	Putative membrane-bound redox modulator
Butane-diol	*cspC* ^c^	10.8	23.3 (3E−5)	Stress protein, member of the CspA-family
	*ydaE* ^c^	8.3	ND	Rac prophage; conserved protein
	*ydhU*	6.4	23.5 (1E−5)	Putative cytochrome b subunit of YdhYVWXUT oxidoreductase
	*yebG*	6.3	−26.1 (3E−5)	DNA damage-inducible protein regulated by LexA
	*iscR*	4.3	NS	Isc operon transcriptional repressor
	*ymfL*	4.1	NS	e14 prophage; putative DNA-binding transcriptional regulator
Furfural	*ydaE* ^c^	8.2	−31.1 (1E−4)	Rac prophage; conserved protein
	*ydaG* ^c^	7.9	ND	Rac prophage; uncharacterized protein
	*ydhU*	7.2	−22.8 (4E−4)	Putative cytochrome b subunit of YdhYVWXUT oxidoreductase
Decanoic acid	*dusB* ^c^	18.3	ND	tRNA-dihydrouridine synthase B
	*greA* ^c^	17.4	ND	Transcript cleavage factor
Geraniol	*acrR* ^c^	303.4	ND	Transcriptional repressor
	*yfeH* ^c^	91.1	ND	Putative inorganic ion transporter
Acetate	*acrR* ^c^	6.5	7.5 (9E−3)	Transcriptional repressor
	*azuC* ^c^	5.1	ND	Acid-inducible small membrane-associated protein
	*ydaE*	4.3	17.0 (5E−4)	Rac prophage; conserved protein
	*yebG*	3.2	NS	DNA damage-inducible protein regulated by LexA
	*cspC*	2.8	36.8 (2E−2)	Stress protein, member of the CspA-family
	*ydhU*	2.3	NS	Putative cytochrome b subunit of YdhYVWXUT oxidoreductase
Itaconic acid	*fnrS* ^c^	7.5	ND	sRNA anaerobic regulator
	*tfaX* ^c^	7.2	ND	DLP12 prophage; predicted protein
	*rpsT*	7.0	−22.2 (6E−5)	30S ribosomal subunit protein S20
	*tam*	5.3	−23.3 (3E−4)	Trans-aconitate methyltransferase
	*racC*	4.4	NS	Rac prophage; uncharacterized protein
Levulinic acid	*azuC* ^c^	7.0	ND	Acid-inducible small membrane-associated protein
	*rpsT* ^c^	6.6	ND	30S ribosomal subunit protein S20
Succinic acid	*marB* ^c^	8.6	19.5 (8E−3)	Mar operon regulator
	*insH1* ^c^	6.1	ND	IS5 transposase and trans-activator
	*brnQ*	4.1	39.8 (7E−4)	Branched-chain amino acid transport system 2 carrier protein
	*cpxA*	1.6	37.0 (3E−3)	Sensory histidine kinase. Two-comp. regulatory system with CpxR
Serine	*cpxA* ^c^	32.3	ND	Sensory histidine kinase. Two-comp. regulatory system with CpxR
	*marB* ^c^	10.3	NS	Mar operon regulator
Threo-nine	*ydiN* ^c^	245.7	13.6 (3E−2)	MFS transporter superfamily protein
	*brnQ* ^c^	61.7	60.2 (4E−4)	Branched-chain amino acid transport system 2 carrier protein
	*dusB*	12.5	59.0 (1E−4)	tRNA-dihydrouridine synthase B

*NS* not significant. Tested gene mutant displays no significant change in growth rate

*ND* not determined. Gene mutant not experimentally tested

^a^Keio collection *E. coli* BW25113 gene deletion mutants

^b^Only Tn-seq transposon mutants with top-two fold changes and/or tested Keio mutants are included

^c^Gene with a top-two fold change in the particular condition

The cellular function of the significant genes is varied not just for those in Table 3 but also among all significant genes. Some of the beneficial insertions could be expected, such as *rob* for butanol, *marB* for butyrolactone and *acrR* for geraniol. These are involved in the regulation of the *mar*-*sox*-*rob* regulon which is known to be involved in e.g. organic solvent tolerance [44]. Many other insertions are not intuitively beneficial and many have unknown functions. Among the detrimental insertions are multiple genes encoding cytochrome bo (*cyo*), NADH oxidoreductase (*nuo*) and LPS synthesis (*waa*) enzymes in geraniol (*cyo*) and butanol and decanoic acid conditions (*cyo*, *nuo* and *waa* genes).

Optimally, gene deletions improving fitness towards not just one chemical stress, but multiple chemical stresses could be revealed using Tn-seq, and some transposon mutants also appear in several conditions within the top-two mutants with highest fold changes, i.e. *marB* (Byr, Ser, Suc), *ydaE* (Diol, Furf), *acrR* (Ger, Ace) and *azuC* (Ace, Lev). Even though *marB* and *acrR* repress genes related to e.g. organic solvent tolerance their inactivation is also beneficial in acid and amino acid conditions. Additionally the deletion of *azuC*, encoding an acid inducible protein, could be relevant for improving acid tolerance because its inactivation improves fitness in two acid conditions. Other genes were also found to be enriched in insertions across multiple conditions as well (Table 4). However, many of the genes presented in Table 4 generally have lower fold changes, as is displayed by the fact that only one, *ydaE*, is present among the genes with top-two fold changes. Consequently, these genes and their related processes could be examples of smaller yet general chemical tolerance mechanisms.

**Table 4:** Transposon mutants predicted to result in improved fitness across multiple conditions

Gene	Quantity	Chemicals^a^												Function
		But	Byr	Diol	Furf	Ace	Ita	Lev	Suc	Deca	Ger	Ser	Thre	
*ydhU*	10	x	x	x	x	x	x	x	x	x	x			Putative cytochrome b subunit of oxidoreductase
*yebG*	9	x	x	x	x	x		x	x	x	x			DNA damage-inducible protein regulated by LexA
*mngA*	8	x	x	x	x	x	x	x		x				2-*O*-a-mannosyl-d-glycerate PTS permease
*gmr*	7	x		x	x	x	x	x		x				Cyclic-di-GMP phosphodiesterase
*iscR*	7	x		x	x	x		x		x	x			Isc operon transcriptional repressor
*pnp*	7	x		x	x	x		x		x	x			Polynucleotide phosphorylase/polyadenylase
*rlmH*	7	x		x	x	x	x	x		x				23S rRNA pseudouridine methyltransferase
*ycjW*	6	x		x	x	x		x		x				LacI family putative transcriptional repressor
*ydaE*	6	x		x	x	x		x		x				Rac prophage
*yffO*	6			x	x	x		x		x	x			CPZ-55 prophage
*ymfL*	6	x		x	x	x		x		x				e14 prophage, putative transcriptional regulator

^**a**^x denotes if gene transposon mutant is significantly enriched in the particular condition

Tn-seq and RNA-seq are two different approaches for investigating the chemical stress in *E. coli* and it is therefore relevant to compare the results of these two methods. Comparison reveals that there is no general overrepresentation of Tn-seq significant genes among the RNA-seq significant genes. The average percentage of total RNA-seq significant genes is 26.7 % while among Tn-seq significant genes 27.3 % are also significant in RNA-seq, corresponding to a P value of 0.63 for a paired t test comparison. Furthermore, the fold change direction between the two methods is randomly distributed with 13.4 % of Tn-seq significant genes having the same direction in RNA-seq and 14 % with an opposite direction in RNA-seq (paired t test P value 0.85). The distributions are visualized by box-plots in Additional file 3. Consequently, there is no general trend of overlap in identified genes between these two methods.

## Discussion

In the present study two strategies were pursued to elucidate chemical tolerance mechanisms in *E. coli*. One entailed finding shared features of the transcriptional chemical stress response that can be applied as a guide for rational tolerance engineering towards multiple chemicals while the other involved transposon mutant library screening for genetic tolerance determinants. Understanding the transcriptional response of *E. coli* can provide clues to enable rational tolerance engineering.

**Fig. 5:**
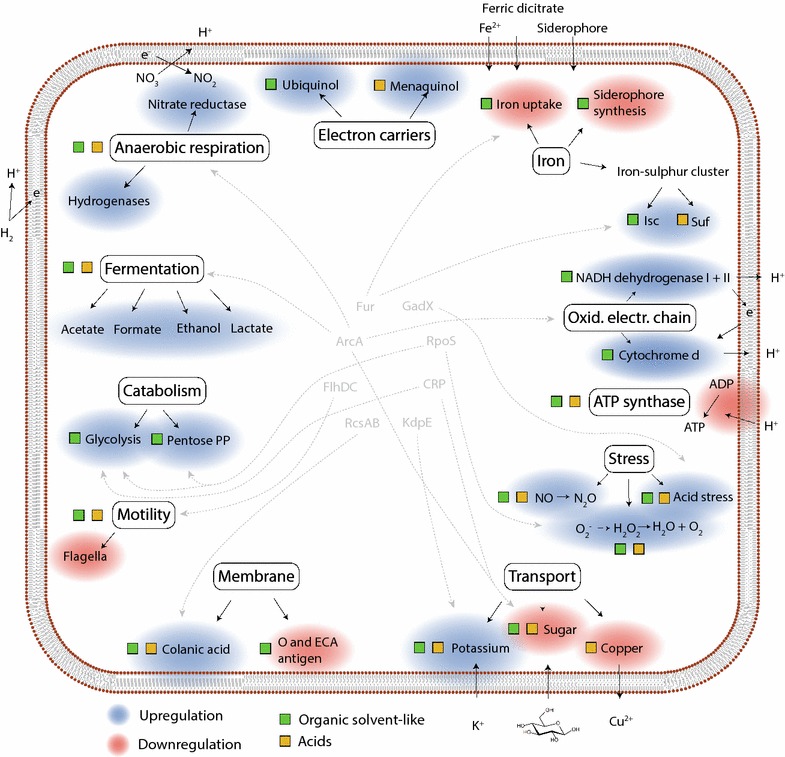
Selected cellular functions with differential gene expression in multiple organic solvent and/or acid conditions. *Blue* highlighting signifies upregulation and *red* highlighting signifies downregulation of genes associated with the particular function. *Colored squares* denote gene expression changes present in at least 3 out of 4 organic solvent (*green squares*) or acid (*orange squares*) conditions. Gene regulators are depicted in *grey letters* with *arrows* indicating functions on which they exert a regulatory effect

Widely altered gene expression was observed when cells were exposed to the 12 selected chemicals. This was especially true for organic solvent (butanol, butyrolactone, 1,4-butanediol, furfural) and acid (acetate, itaconic, levulinic and succinic acid) chemical stresses and these conditions share expression changes in many general functional categories such as energy metabolism, iron metabolism as well as transport and stress mechanisms (Fig. 5). Notably, the upregulation of anaerobic respiration and fermentation genes constitute a general feature of organic solvent and acid conditions. The functional outcome of increased anaerobic respiration is periplasmic proton generation and thus a likely increase of the proton gradient for energy generation [45]. The upregulation of electron transport chain components for organic solvent conditions should provide the same effect, while the upregulation of glycolysis and pentose phosphate pathway genes would also increase energy generation. This response during organic solvent stress could be due to an increased cellular maintenance energy caused by these chemicals.

Many of the genes of anaerobic respiration and energy metabolism are known to be regulated by ArcA and previous studies on butanol and isobutanol stress in *E. coli* [12, 13] also identified ArcA as involved in the stress response. Correspondingly, the regulatory footprint of changed ArcA activity was evident here during butanol stress, and while the ArcA regulon was enriched in the other organic solvent conditions, the footprint was less apparent. The physiological cause during isobutanol stress was hypothesized to be quinone malfunction due to membrane perturbation leading to ArcA activation [13]. The same phenomenon appears to be present for the butanol condition based on the differential expression of the ArcA regulon. The other three organic solvent conditions also had increased expression of oxidative electron transport chain genes, but only few ArcA-regulated genes with differential expression in the expected direction. Thus the oxidative electron chain gene upregulations here are possibly not ArcA mediated and could instead be a more direct effect of membrane perturbation and possible quinone malfunction. The overall likely effect of the respiration and energy metabolism gene upregulations for both organic solvent and acid conditions is an increase in the proton gradient. For organic solvent conditions this could be a means to counter the reduced membrane potential caused by membrane perturbation while for acid conditions it could counter reduced proton motive force caused by cytoplasmic acidification [46]. The presence of weak organic acids in the medium can lead to intracellular acidification by diffusion of undissociated acid [47] while the reduced or possibly lost proton gradient for organic solvents would correspond to a lower intracellular pH that is likely to mediate the observed acid stress response.

For organic solvent conditions iron metabolism is also affected (Fig. 5), where iron uptake systems are downregulated likely by Fur activation. This was also previously observed during isobutanol stress [13] and explained by a reduced occurrence of the Fenton reaction, as quinone failure causes less superoxide to be created through respiration. This ultimately leads to increased Fe^2+^ concentration and Fur activation resulting in iron uptake-system downregulation. Consequently reduced iron (Fe^3+^) availability is a likely effect of organic solvent stress. Fur activation could also provide an explanation for the upregulation of the isc iron–sulphur cluster assembly system. The suf iron–sulphur cluster assembly system which is upregulated in acid conditions can conversely be induced by oxidative stress [48]. The observed upregulation of *sodC* and *katE* genes in these conditions supports the induction of oxidative stress in acid conditions.

Apart from iron transport, other transport mechanisms are affected, among these sugar transport and copper transport. CRP is a known regulator of the sugar transport genes and could mediate this. However, it could also be a growth rate related effect in the stress conditions, leading to a reduced glucose uptake demand, although this does not correspond well with the upregulation of glycolysis in organic solvent conditions. The *cusABCF* copper transport genes are downregulated in acid and amino acid conditions. CusR is the cognate regulator responding to copper concentration while the Cus transporter mediates periplasmic Cu^+^ efflux, having a role in alleviating copper toxicity under mainly anaerobic conditions [49]. The underlying reason for the downregulation of the transporter here is unclear. Upregulation of colanic acid synthesis genes is present for organic solvent and acid conditions. Colanic acid is a negatively charged extracellular polysaccharide loosely associated with the outer membrane and is required for biofilm structure in *E. coli* [50, 51]. Apart from this, it is generally only produced during stressful conditions providing an explanation of its upregulation here. It has been shown to confer increased acid and osmotic stress resistance [52–54] and could thus contribute towards increased tolerance.

Altered activity of some of the functions shown in Fig. 5 was previously associated with increased fitness during butanol stress. Increased external iron concentration and increased expression of iron transport genes (*fepA, feoA*) or siderophore synthesis genes (*entC, entD*) improved *E. coli* butanol tolerance [26, 28, 55]. Consequently, increasing iron availability appears to improve fitness. As mentioned, the Fur-mediated downregulation of iron-uptake mechanisms likely leads to intracellular iron depletion. As this is the case for all organic solvent conditions, this strategy should therefore prove advantageous under conditions other than butanol stress. Genes related to another main function in Fig. 5, anaerobic respiration, have also been identified. Upregulation of nitrate reductase two genes were found in a CRP engineered strain with improved butanol tolerance [32] while overexpression of *hyaF* also improved fitness during isobutanol stress [23]. Accordingly, a more systematic approach towards increasing respiration could increase organic solvent tolerance in *E. coli*, likely by increasing energy generation through an improved proton gradient. A potential risk is a lower yield in a production scenario, however this might be outweighed by improved survival or cellular state. Another strategy could be increasing membrane protein expression to account for the apparent membrane effects during many chemical stresses, analogous to the method of improving osmotic stress tolerance [56]. Moreover, the CRP engineered strain with improved isobutanol tolerance displayed increased expression of acid stress resistance genes (glutamate decarboxylase and acid fitness island) [32], further implicating these in butanol tolerance. Overall, the above findings substantiate that several of the functions outlined in this study are targets for rational tolerance engineering. Apart from those mentioned it includes the identified significantly enriched regulators, their regulon members together with the presented functional gene categories.

The enriched and depleted genes obtained by Tn-seq provide a list of gene deletions that potentially confer either increased or reduced fitness in a given chemical stress. Although Tn-seq involves mutant generation by transposon insertion, the functional outcome is generally equivalent to a gene deletion as only insertions within protein coding regions are included during the analysis. There are in total 294 enriched gene-insertions and 336 depleted gene-insertions in the 12 conditions, and thus at least 294 gene deletions that potentially increase the fitness in one or more chemical stress conditions. A number of these have been experimentally verified and display a range of growth rate increases from 8 to 60 %. In general the genes belong to diverse functional classes and a large fraction have unknown or scarcely known function. Yet, some (*marB, rob, acrR*) relate to the Mar-Sox-Rob regulon that is involved in e.g. organic solvent tolerance and superoxide resistance and is regulated by the three transcription factors MarA, SoxS and Rob [44, 57, 58]. The enriched insertions in *marB* (Byr, Furf, Deca, Suc, Ser) would lead to upregulation of *marA* and hence the Mar-Sox-Rob regulon [59]. Interestingly it is not the principal *marA* repressor, *marR,* which is enriched. In contrast, the enrichment of *rob* in the butanol condition abolishes any *rob*-mediated activation of the regulon, corresponding with the finding of increased isobutanol tolerance for *acrAB* and *marCRAB* deletion mutants [13]. The *acrAB* and *tolC* genes encoding an efflux pump are part of the Mar-Sox-Rob regulon [60]. Another transcriptional repressor of *acrAB* is AcrR which was found to be enriched in decanoic acid, geraniol and acetate conditions, indicating that upregulation of this efflux pump confers increased tolerance here. This was previously shown for geraniol [61] and loss-of-function of *acrAB* has also been implicated in reduced tolerance toward decanoic acid and other free fatty acids [20]. Although the efflux pump is associated with increased organic solvent tolerance (e.g. hexane and octane), *acrR* is not enriched in any of the four organic solvent conditions here, likely a result of their much lower octanol–water partition coefficient as compared to *n*-alkanes, decanoic acid and geraniol.

Among the enriched genes with high fold changes or presence in multiple conditions are also many related to RNA or DNA processes. Examples of the enriched genes include *srmB* (helicase, ribosomal association), *cspC* (antiterminator activity, thought to stabilize *rpoS* mRNA), *rraA* (Rnase E inhibitor), *dusB* (5,6 dihydrouridine modification in tRNA, possible ROS and stress regulator), *greA* (release stalled polymerase), *rpsT* (S20 ribosomal protein), *pnp* (mRNA degradation, minimizes oxidatively damaged RNA) and *rlmH* (23S rRNA pseudouridine methyltransferase). For most, the beneficial effect is not immediately intuitive, however many of these gene deletions would be expected to result in broad effects on transcription and translation processes. It is likely that many chemicals in this study elicit defects in transcription and translation, as was found for ethanol stress [62]. The inactivation of the *brnQ* gene provides the largest growth rate increase (60 %) and is enriched in threonine, succinic acid and butyrolactone conditions, although the latter could not be experimentally verified. It constitutes a branched chain amino acid transporter (LIV-II). Threonine is known to be a substrate of the LIV-I transport system [63] and based on the present findings possibly the LIV-II system as well, as its inactivation increases fitness during threonine stress. The most depleted gene insertion (*ompA*) is depleted in nine conditions (organic solvents, three acids and two membrane-intercalating conditions) revealing its relevance during chemical stress. Its overexpression could possibly provide a general tolerance mechanism towards many chemicals.

To be certain of the fitness effects of specific gene deletions, further experimental testing is required. Yet, by testing the fitness effect by measuring growth rate changes for 35 gene deletion mutants enriched in Tn-seq the majority of these conferred a fitness increase. In reality the proportion of fitness increasing gene deletions could be higher as a growth rate assay is a relatively insensitive assay. Employing a survival or competition assay might provide additional experimental verification. Further, in a production scenario a mere reduction of stress, without an effect on growth rate, could improve production. Additionally, as the Keio collection was employed for experimental verification, strain differences exist between the Tn-seq (K-12 MG1655) and growth rate experiments (K-12 BW25113). Some fitness enhancing mutations are likely not shared between these two genotypes.

A comparison of the RNA-seq and Tn-seq results showed no general overrepresentation of Tn-seq significant genes among the RNA-seq significant genes nor the presence of any direction-wise overrepresentation. This is not unexpected as each method provides information on different parameters, specifically transcriptional change or gene fitness. Further the chemical stresses represented here do not generally represent natural stresses encountered by *E. coli* in the environment, except for acetate and to some extent the acids. Although only 25 % of Tn-seq significant genes overlapped with corresponding RNA-seq significant genes for acetate conditions, most were enriched in the same direction. The general discrepancy does not imply that the methods provide conflicting results but rather that the two methods are complementary and each provide different levels of insight and targets for tolerance improvement. Consequently utilizing both approaches will increase the prospects for successful tolerance engineering.

## Conclusions

The selection of 12 chemicals in this study enables the identification of general targets that can improve tolerance towards not just a single but a suite of chemicals. This potentially also includes chemicals with similar properties to those presented here. The transcriptional response of *E. coli* towards the 12 chemicals identified several functions that could be targeted by rational engineering, among these anaerobic respiration and fermentation genes for organic solvents and acids. In particular, improving the proton motive force for maintaining efficient energy utilization is an important parameter in both conditions. Further, engineering of stress responses such as oxidative stress and acid resistance systems could improve tolerance, while for organic solvents iron metabolism provides another engineering target. Some of the regulons with perturbed expression for several chemical stresses include ArcA, CRP, RpoS, OmpR, Fur and GadX and altering these could likewise enhance beneficial or eliminate detrimental expression changes. In the other approach of genome-wide screening of transposon mutants, the substantial number of enriched mutants (294) that were identified provides a hit-list for further study. Some of the target genes, such as those related to the Mar-Sox-Rob regulon, are known to be stress-related, however those associated with transcription and translation processes have so far received less attention in a chemical stress context and warrants further examination. Overall the two approaches have yielded different targets and therefore provide complementary information for engineering improved tolerance.

## Methods

### Bacterial strains and growth conditions

The *Escherichia coli* K-12 MG1655 strain was employed for transcriptional profiling while the strain background for Tn-seq experiments was *Escherichia coli* K-12 MG1655 Δ*hdsR*. The growth medium was M9 medium [64] with 0.2 % glucose with added trace elements and vitamins, as described previously [65]. For transcriptome chemical stress experiments cells were grown overnight in M9 medium and diluted to an OD_600_ of 0.05 in 25 mL M9 medium in 250 mL baffled shake flasks. After growing to ~OD_600_ 0.9, while the cells were still growing exponentially, 25 mL M9 medium with chemical stressor was added to the medium (in effect halving the cell density) and cells were grown for 1 h before harvest. Control cells without chemical stressor in the added 25 mL of M9 medium were grown for approximately 0.5 h to reach the same OD_600_, 0.6–0.7, as the chemically stressed conditions. Biological triplicates were performed for all conditions and the employed chemicals were sodium acetate (Sigma, S8750), butanol (Sigma, 281549), 3-hydroxy-butyrolactone (TCI Chemicals, H0939), 1,4-butanediol (Merck, 801534), decanoic acid (Sigma, W236403), furfural (Sigma, 185914), geraniol (TCI Chemicals G0027), itaconic acid (Sigma, I29204), levulinic acid (Sigma, L2009), l-serine (Sigma, S4311), succinic acid (Sigma, S9512) and l-threonine (Sigma, T8441). Where appropriate (itaconic acid, levulinic acid, succinic acid conditions) the medium was neutralized to pH 6.8 with NaOH.

### Isolation and processing of RNA

RNA isolation and processing was performed as previously described [66]. Briefly, cells were harvested by adding 10 mL of cell culture to 2 mL ethanol containing 5 % phenol, followed by pelleting of cells by centrifugation and freezing at −80 °C. Subsequently cell pellets were lysed with 1 mg/mL lysozyme for 5 min and RNA extracted using Trizol and chloroform. For DNA removal each sample was treated with 40 units DNase I for 30 min. The purity and quality of RNA was verified with spectrophotometer and Bioanalyzer (Agilent Technologies). Extracted RNA was depleted of 23S, 16S and 5S rRNAs by subtractive hybridization using the MICROBExpress kit (Life Technologies) and adding an HPLC purified custom Capture Oligo specific for 5S rRNA (5′-AAAAAAAAAAAAAAAAAAGCGTTTCACTTCTGAGTTCGGCA-3′). Transcript libraries were prepared using the TruSeq RNA Sample Preparation Kit v2 (Illumina) with minor modifications. The protocol was initiated at the Elute, Prime, Fragment step using 100–400 ng of rRNA-depleted RNA and followed until the completion of the Enrich DNA Fragments step. Libraries were quantified using a Qubit Fluorometer and size distribution assessed on a Bioanalyzer. Libraries were single-end sequenced on an Illumina Hi-Seq 2000 at BGI-Europe.

### Transcriptome data analysis

Obtained reads were trimmed employing Trimmomatic (default settings) [67] in order to remove low-quality read regions. Reads were mapped to the *E. coli* K-12 MG1655 genome (NC_000913.2) using CLC Genomics Workbench with default settings and read counts per gene extracted. Subsequently TMM normalization [68], a scaling normalization method employing weighted trimmed mean of the log expression ratios, was utilized. Differential expression analysis was performed with the edgeR R statistics package [68], regarding counts as a negative binomial distribution and data fitted to generalized linear models. Default parameters were employed and genes with FDR-values below 0.01 were defined as significantly differentially expressed. Heat map and hierarchical clustering employing the Pearson correlation as distance measure was performed with the R packages bioDist and gplots using normalized log(2)-transformed expression values. Regulon analysis was performed using the gene set analysis pipeline from the piano R package [42]. The GSA method was set to median and fold change direction and P values from differential expression analysis were employed. Regulon classifications were obtained from RegulonDB [69]. Regulon sets with non-directional adjusted P values below 0.05 were designated as significant.

### Transposon mutant library growth selection

The transposon (Tn5) mutant library was constructed as previously described [70]. Briefly, *E. coli* K-12 MG1655 Δ*hdsR* was transformed with the transposase-transposon complex from the EZTn5 R6 Kγori/KAN-2_transposome kit (Epicentre, Madison, WI). Approximately 60,000 transposon mutans were generated in this library. For chemical stress selection, cryogenic library stock was grown at 37 °C in 50 mL M9 with 25 μg/mL kanamycin in a 250 mL shake flask. Before entry into stationary phase the culture was washed twice and inoculated in 50 mL M9 medium with or without chemical stressor to an OD_600_ of 0.05 and grown at 37 °C with shaking at 250 rpm. Concentrations of chemicals that lead to a doubling time of approximately 3 h, as compared to 1 h for the control, were chosen. Concentrations were: butanol 87 mM; butyrolactone 79 mM; butanediol 791 mM; furfural 12 mM; decanoic acid 16 mM, geraniol 3 mM; acetate 103 mM; itaconic acid 361 mM; levulinic acid 198 mM; succinic acid 381 mM; serine 67 mM; threonine 118 mM. To avoid entry into stationary phase, cultures were transferred at an OD_600_ of 1 into fresh M9 medium with or without stressor to an OD_600_ of 0.05. Cells were grown up to an OD_600_ of 1, harvested, centrifuged and cell pellets stored at −20 °C. Additionally, the preculture used for inoculation was subjected to the same procedure, serving as an initial mutant distribution reference.

### Tn-seq DNA library generation and data analysis

DNA libraries were prepared for sequencing as previously described [70]. Briefly, genomic DNA was extracted from cell pellets using the PureLink genomic DNA minikit (Life Technologies) and 2–3 μg DNA sheared in a Covaris E220 ultrasonicator. DNA fragments were subjected to end repair (NEBNext end repair module), dA-tailing (Klenow fragment exo^−^), ligated (NEBNext quick ligation module) with Illumina TruSeq adapters and PCR enriched using adapter and Tn5-specific biotinylated primers. Biotinylated PCR products were affinity captured using Dynal MyOne streptavidin C1 beads (Invitrogen). Single-stranded DNA was generated with 0.15 M sodium hydroxide, quantified by qPCR (Kapa Library Quantification kit) and size distribution determined (Agilent RNA 6000 Pico kit, Agilent 2100 Bioanalyzer). For each condition duplicate biological experiments were performed followed by sequencing, on an Illumina MiSeq. For itaconic acid only one sample was utilized in subsequent steps as one replicate had low sequencing depth. Reads were trimmed to 67 bp (containing 25 bp genomic sequence) and sequencing data analyzed using the ESSENTIALS pipeline [71] with the following parameters deviating from default settings: 150,000 library size; 3 nucl. barcode mismatch; 20 bp genomic sequence match. Gene-level insertion counts were normalized using TMM normalization and P values adjusted by Benjamini-Hochberg correction. Genbank version NC_000913.3 of *E. coli* K-12 MG1655 was employed. Selection pressure purely originating from the M9 medium was accounted for with the control condition (without chemical stressor). Fold changes of significantly enriched insertion mutants during stress (stress vs. control) were multiplied with the control condition fold change (control vs. initial pre-culture mutant distribution), if the gene was simultaneously significantly depleted in the control condition compared to the initial distribution. After division only insertion mutants with resulting fold change values above 1 were defined as significantly enriched during stress conditions (Eq. 1).

1 $$Stress\, FC*Control \, FC = \frac{stress \, count}{control \, count}* \frac{control \,count}{initial \,count} > 1$$

Thus only insertion mutants enriched compared to both the control condition and the initial distribution were included. The same procedure was applied for depleted insertion mutants.

### Growth experiments of gene deletion strains

Keio collection (K-12 BW25113) [43] gene deletion strains were employed for experimental verification of Tn-seq derived target deletions. Strains were grown overnight in M9 with trace elements and vitamins. An equivalent volume of fresh M9 medium was added to overnight culture and cells grown for at least 1 h to exit stationary phase, followed by inoculation into fresh M9 medium with trace elements, vitamins and with or without stressor to an OD_600_ of 0.05. A 1.4 mL culture volume was grown in Flowerplates with 1000 rpm shaking at 37 °C in a Biolector (m2p-labs). Growth rates were determined from at least triplicate cultures and significant differences between deletion strains and wildtype strain determined by one-tailed t test, P < 0.05.

## Additional files

10.1186/s12934-016-0577-5 Differential gene expression values for each chemical stress condition compared to the control condition.

10.1186/s12934-016-0577-5 Enrichment or depletion of gene transposon mutants under chemical stress conditions.

10.1186/s12934-016-0577-5 Comparison of distributions of significant genes between RNA-seq and Tn-seq.

## Abbreviations

FFA: free fatty acids
Tn-seq: transposon sequencing
Byr: hydroxy-γ-butyrolactone
Furf: furfural
Ita: itaconic acid
Lev: levulinic acid
Ser: l-serine
Suc: succinic acid
Thre: threonine
Ace: acetate
But: butanol
Diol: 1,4-butanediol
Ger: geraniol
Deca: decanoic acid
ECA: enterobacterial common antigen
CRP: catabolite repression protein
